# High expression of long intervening non-coding RNA *OLMALINC* in the human cortical white matter is associated with regulation of oligodendrocyte maturation

**DOI:** 10.1186/s13041-014-0091-9

**Published:** 2015-01-10

**Authors:** James D Mills, Tomas Kavanagh, Woojin S Kim, Bei Jun Chen, Paul D Waters, Glenda M Halliday, Michael Janitz

**Affiliations:** School of Biotechnology and Biomolecular Sciences, University of New South Wales, Sydney, NSW 2052 Australia; Neuroscience Research Australia, Sydney, NSW 2031 Australia; School of Medical Sciences, University of New South Wales, Sydney, NSW 2052 Australia; Present address: Garvan Institute of Medical Research, Sydney, NSW 2010 Australia

**Keywords:** Long intervening non-coding RNA, *OLMALINC*, Human brain, Frontal cortex, White and grey matter, Antisense RNA

## Abstract

**Background:**

Long intervening non-coding RNAs (lincRNAs) are a recently discovered subclass of non-coding RNAs. LincRNAs are expressed across the mammalian genome and contribute to the pervasive transcription phenomenon. They display a tissue-specific and species-specific mode of expression and are present abundantly in the brain.

**Results:**

Here, we report the expression patterns of oligodendrocyte maturation-associated long intervening non-coding RNA (*OLMALINC*), which is highly expressed in the white matter (WM) of the human frontal cortex compared to the grey matter (GM) and peripheral tissues. Moreover, we identified a novel isoform of *OLMALINC* that was also up-regulated in the WM. RNA-interference (RNAi) knockdown of *OLMALINC* in oligodendrocytes, which are the major cell type in the WM, caused significant changes in the expression of genes regulating cytostructure, cell activation and membrane signaling. Gene ontology enrichment analysis revealed that over 10% of the top 25 up- and down-regulated genes were involved in oligodendrocyte maturation. RNAi experiments in neuronal cells resulted in the perturbation of genes controlling cell proliferation. Furthermore, we identified a novel *cis*-natural antisense non-coding RNA, which we named *OLMALINC-AS*, which maps to the first exon of the dominant isoform of *OLMALINC*.

**Conclusions:**

Our study has demonstrated for the first time that a primate-specific lincRNA regulates the expression of genes critical to human oligodendrocyte maturation, which in turn might be regulated by an antisense counterpart.

**Electronic supplementary material:**

The online version of this article (doi:10.1186/s13041-014-0091-9) contains supplementary material, which is available to authorized users.

## Background

There is a growing comprehension that the majority of the transcribed genome does not code for proteins but comprises a variety of non-coding RNAs of different properties, such as length, as well as functionality in transcriptional and epigenetic control [1]. In particular, long non-coding RNAs (lncRNAs) seem to be a very recent evolutionary development, and this is supported by the observation that primates, and humans in particular, express most of the currently characterized lncRNAs. A subcategory of lncRNAs, termed long intervening non-coding RNAs (lincRNAs), show a tissue-specific mode of expression and substantially contribute to the pervasive transcription that is observed in the mammalian genome. LincRNAs are defined as intervening (relative to the current gene annotations) transcripts that are longer than 200 nucleotides in length and lack protein-coding capacity [2,3].

The role of lincRNAs is thought to be in gene regulation, and in the brain it has been proposed that non-coding RNAs may play a role in regulating axon myelination in WM and glial cell differentiation [4]. It is thought that lincRNAs act as scaffolds to allow for epigenetic changes to occur within the genome, such as increasing or decreasing mRNA levels through sequestration or stabilization, thus regulating the transcription of target genes. Such a process is of the utmost importance in the development of the complicated array of cells that is observed within the brain.

There is a growing body of evidence that the human brain, as the most complex organ in the body, is the richest source of lncRNAs, reflecting the demand for highly complex control mechanisms for the differentiation and development of numerous cell types, including neurons and oligodendroglia [4]. Indeed, our recent RNA sequencing (RNA-Seq) study has revealed vast differences between the transcriptomes of the GM and WM in the human superior frontal cortex [5]. While not unexpected, given the differences in cell type populations within these two structures, this study has demonstrated that a significant number of annotated and unidentified lincRNAs are expressed at high levels and disparate quantities between GM and WM.

One of the most noticeable examples of differentially expressed lincRNAs between WM and GM was *linc00263* [5]. Compared to most lincRNAs, which remain at low expression levels, i.e. < 2 fragments per kilobase of exon per million fragments mapped (fpkm), *linc00263* was expressed at 16.2 and 71.5 fpkm in GM and WM, respectively [5]. The high levels of expression in WM suggest that *linc00263* may have a functional importance in oligodendrocytes, which constitute the majority of cells in the WM. Here, we validated the differential expression of *linc00263* in GM and WM using RT-qPCR. Furthermore, we performed a comparative analysis of *linc00263* using vertebrate genomes and found that this gene is specific to primates. Finally, RNAi knockdown of *linc00263* in human neuronal and oligodendrocyte cell lines followed by transcriptome sequencing demonstrated that silencing of *linc00263*, which we renamed to *OLMALINC* (oligodendrocyte maturation-associated long intervening non-coding RNA), results in coordinated changes in the expression of genes controlling the cytoskeleton and maturation of glial cells.

## Results

### *OLMALINC* is highly expressed in the white matter of the human frontal cortex

**Figure 1:**
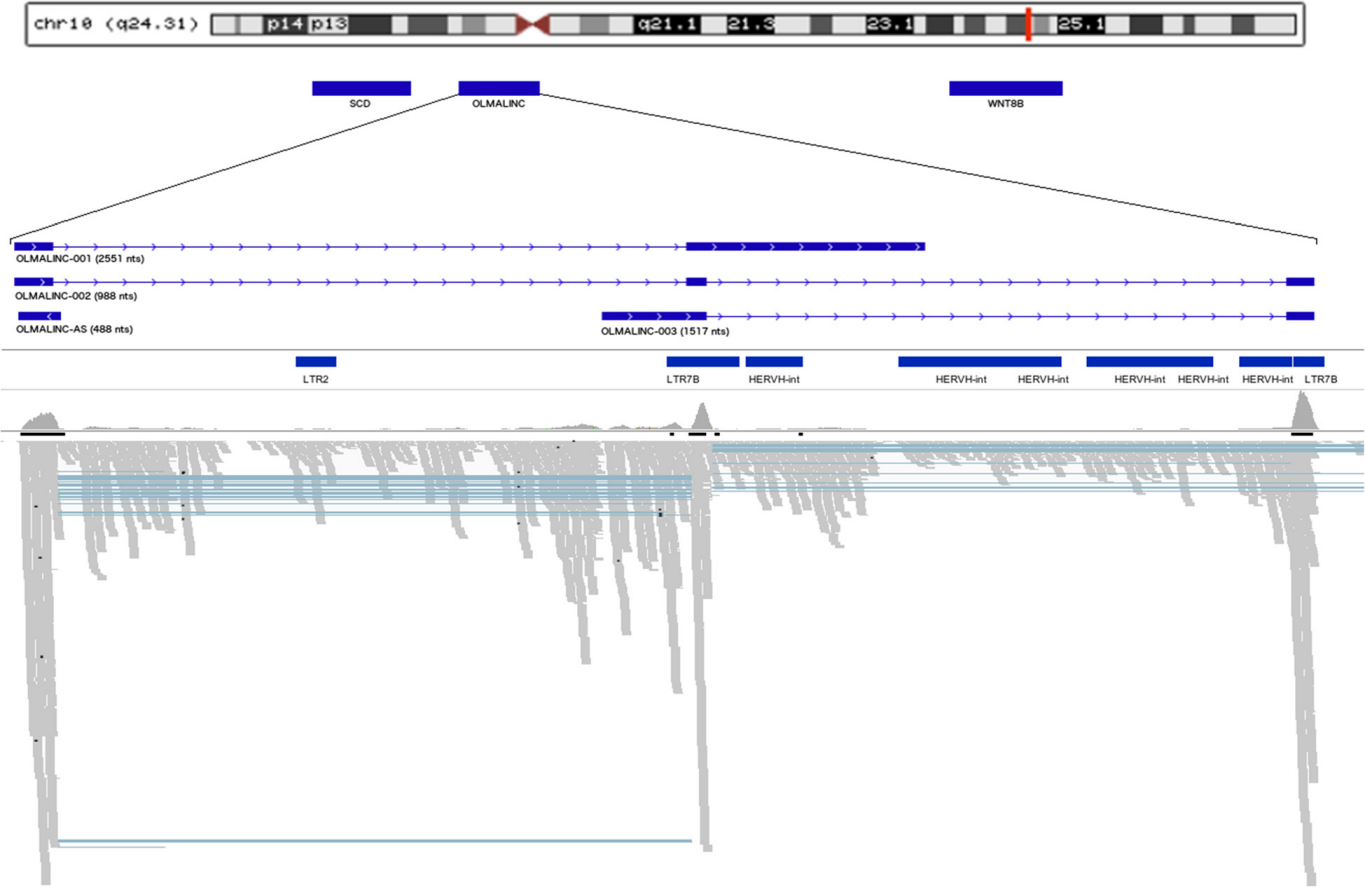
**Genomic context splice of variants and genomic features of** ***OLMALINC*** **.** Track 1 represents the chromosomal positioning of *OLMALINC*. Track 2 shows *OLMALINC* in genomic context. *OLMALINC* is located downstream of the gene stearoyl-CoA desaturase (delta-9-desaturase) (*SCD*) and up-stream of wingless-type MMTV integration site family, member 8B (*WNT8B*). Track 3 is a schematic representation of the exon/intron structure of the *OLMALINC-001, −002* and −*003* isoforms as well as *OLMALINC-AS*. Track 4 is a schematic of the repeat elements that appears in this region of the genome including long terminal repeats LTR2, LTR7B and HERVH-int. A LTR 7B over laps with a large portion of the final exon of *OLMALINC-002* and *OLMALINC-003.* Track 5 show the read alignment in the WM samples carried out by TopHat, demonstrating that the repeat elements have not artificially inflated the RNA-Seq fpkm values.

In our recently published global analysis of the transcriptome of the WM and GM of the human frontal cortex, we detected 4.4-fold overexpression of *OLMALINC* in WM versus GM [5]. The overall expression of *OLMALINC* was 16.2 fpkm in GM tissue and 71.5 fpkm in WM (Additional file 1: Figure S1A). RNA-Seq analysis revealed that *OLMALINC* is expressed as two isoforms (Figure 1). The alignment pattern of RNA-Seq reads in the WM samples shows equal amounts of reads aligning to each exon of *OLMALINC-002* (Figure 1). This read distribution excludes the possibility of the long terminal repeat (LTR) element, that covers exon 3, might artificially inflate the calculated expression level of *OLMALINC*. Neither of the isoforms represented *OLMALINC-001*, which is annotated in Ensembl [Ensembl ID: ENSG00000235823]. The most highly expressed transcript in our comparative analysis represented the NCBI annotated isoform [NCBI ID: NR_026762.1], which for clarity, we called *OLMALINC-002*. The isoform is 988-nucleotides (nts) long, consists of three exons (Figure 1), and is overexpressed 4.5-fold in WM (62.5 fpkm) compared to GM (14 fpkm) (Additional file 1: Figure S1B). Thus, *OLMALINC-002* is a major contributor to the observed overexpression of the *OLMALINC* gene in WM. The second alternatively spliced isoform was not annotated in any of the reference databases. We named this novel isoform *OLMALINC-003*. It is composed of two exons that form a 1517-nts-long transcript (Figure 1). This novel transcript does not encode any protein, as determined by an open reading frame (ORF) finder. *OLMALINC-003* is expressed at residual levels in GM (1.6 fpkm) and 10 fpkm in WM (Additional file 1: Figure S1B).

To confirm the specificity of the short sequence read alignment and assembly of the data, both *OLMALINC* isoforms were reverse transcribed and amplified using isoform specific primers, and the PCR products were Sanger sequenced. Sequence alignment of the annotated isoform and the sequence extracted from the RNA-Seq data for the novel isoform to the reference genome confirmed the sequence specificity of the RT-PCR results (Additional file 2: Figure S2A and B).

**Figure 2:**
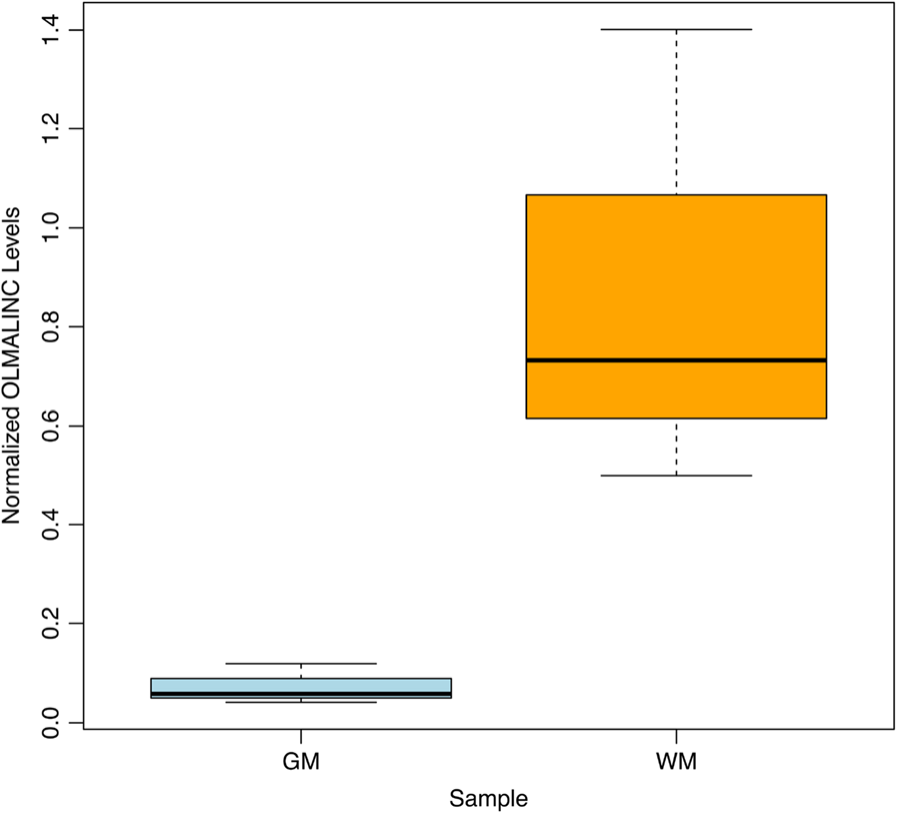
**RT-qPCR validation of the** ***OLMALINC*** **gene expression patterns in GM and WM samples.** This boxplot shows *OLMALINC* was up-regulated 12.05-fold in WM when compared to GM (p-value < 0.05).

Next, we validated the differential expression patterns of the *OLMALINC* using RT-qPCR of independent sets of samples representing the GM and WM of the frontal cortex. The high expression of *OLMALINC*, including both isoforms, was confirmed (Figure 2), with a 12.05-fold higher expression level in WM (p-value< 0.05). The *OLMALINC* primers, used for RT-qPCR, spanned exons 2 and 3 of *OLMALINC-002* and exons 1 and 2 of *OLMALINC-003*. This primer design ruled out the possibility of the high FPKM value attributed to *OLMALINC* by RNA-Seq as a result of additional alignment of reads to the LTR element that covers a large portion of the final exon in both *OLMALINC* isoforms (Figure 1).

### Comparative sequence analysis and expression of *OLMALINC* in mammals

**Figure 3:**
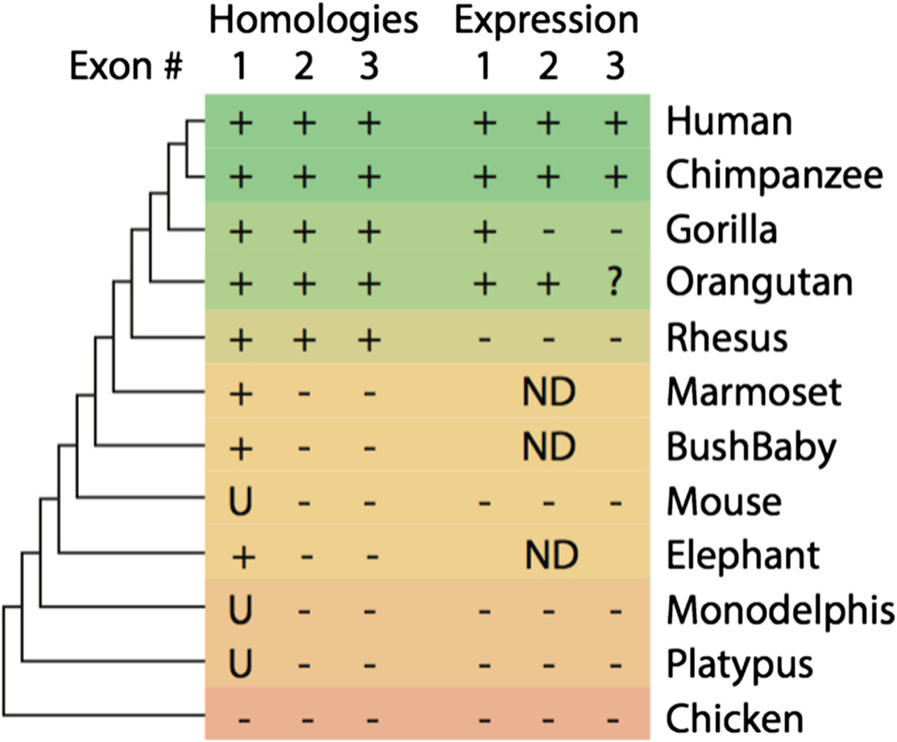
**Summary of homology and expression of** ***OLMALINC*** **(UCSC uc001kqz.4) exons in representative vertebrate genomes.** Homology and expression of all three exons is only detected in human and chimpanzee. Exons 2 and 3 are not detected outside of old world monkeys. There is low expression of exon 3 in orangutan. Outside of great apes there is no expression of any exon. ND = no data. U = homology was only detected in the UCSC genome browser and not by any other method (blastn/blat/dotplots). - = no expression detected. + = expression detected. ? = low levels of expression detected.

Sequence conservation level of *OLMALINC* locus was analyzed using blastn searches, DNA dot plots (https://www.sanger.ac.uk/resources/software/seqtools/) and chain blastz alignments on the UCSC genome browser (https://genome.ucsc.edu/). In primates, homology was detected to all human exons in chimpanzee, gorilla, orangutan and rhesus monkey. In bushbaby, marmoset, mouse, elephant, opossum and platypus homology was only observed with human exon 1 (Figure 3), and in each case in the same genomic context (downstream of the *SCD* gene). The UCSC phyloP base-wise conservation across 100 vetebrates shows a peak in conservation 5′ of *OLMALINC-001* exon 1, suggesting a possible conserved promoter region (Additional file 3: Figure S3).

Analysis of published RNA-Seq data [6,7] revealed expression of all exons in chimpanzee and orangutan brain, although relative expression of exon 3 was lower than in human (Additional file 4: Figure S4). In gorilla brain, only expression of exon 1, and neighboring regions (perhaps the antisense transcript), was detected. RNA-Seq reads did not map to expected exons in any other species examined (Additional file 4: Figure S4), suggesting that *OLMALINC-001* is not expressed outside of great apes. This information is summarized in Figure 3.

### *OLMALINC* expression profiles in non-brain tissues

**Figure 4:**
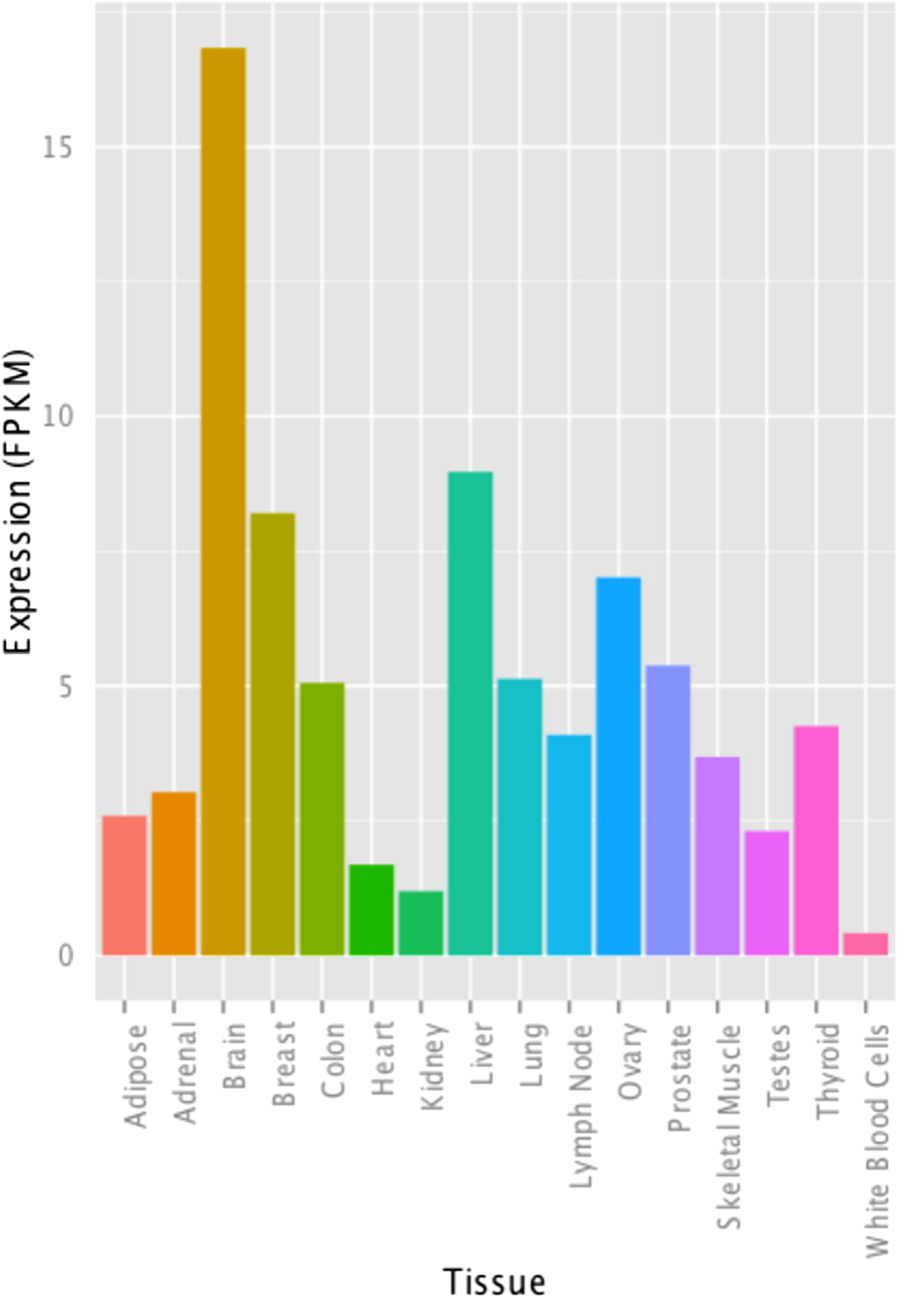
**Comparative analysis of the** ***OLMALINC*** **expression levels in brain and 15 other human tissues.** The bar graph shows the difference in expression levels of *OLMALINC* in varying tissues compared to the brain. The RNA-Seq datasets used for this analysis were taken from Illumina’s BodyMap2 project. The brain has at least a greater than 1.9x expression level of *OLMALINC* than any other tissue. The y-axis is expression in fpkm.

To determine whether *OLMALINC* was likely to be brain-specific or commonly expressed in all tissues, a meta-analysis was performed. This analysis utilized publicly available RNA-Seq datasets from the Illumina Transcriptome BodyMap 2 project, which included samples from 16 human tissues, including brain, using humans of varying ages and sex. The 15 tissues in the Illumina data sets were compared with the brain transcriptome data. The non-brain tissues comprised liver, kidney, heart, lung, skeletal muscle, breast, adrenal, thyroid, prostate, ovary, testes, adipose, colon, lymph node, and white blood cells. The analysis was carried out using the Tuxedo protocol [8] and showed that brain expression level of *OLMALINC* was higher than the expression levels in any other tissue type (Figure 4 and Additional file 5: Figure S5). Liver, ovary and breast tissue had the next highest levels of *OLMALINC* expression; these levels were approximately half of the *OLMALINC* expression seen in the brain. On average *OLMALINC* was expressed 7.5-fold higher in the brain when compared to other sources of tissue. To validate the expression levels from the Illumina Transcriptome BodyMap 2 project, the expression level of *OLMALINC* was analysed in another publicly available RNA-Seq dataset produced by The Human Protein Atlas project. The tissue types assessed were brain, liver, kidney, skin and bone marrow (Additional file 5: Figure S5). *OLMALINC* was expressed at 3-fold higher level in the brain when compared to liver and on average 20-fold higher than the other tissue types. Usage of this independent set of tissue-specific transcriptome sequence data thus corroborates the pattern seen in the Illumina dataset.

### The *OLMALINC* locus co-expresses cis-natural antisense RNA

Recently, we performed strand-specific RNA-Seq analysis of combined RNA samples derived from the GM and WM tissue from the frontal cortex (Mills et al., unpublished data). Bioinformatics analysis of this dataset revealed the presence of an antisense RNA that mapped to the first exon of the *OLMALINC-002* isoform (Figure 1). This antisense transcript is 488-nts long, consists of one exon and is expressed at 18 fpkm in the whole brain tissue. We named this RNA *OLMALINC-AS* to emphasize its *cis*-natural feature in regard to its overlap with the *OLMALINC* locus.

To validate the sensitivity of the strand-specific RNA-Seq analysis, we amplified and Sanger sequenced a fragment that was unique to the *OLMALINC-AS* transcript. Alignment to the reference genome confirmed expression of the *OLMALINC-AS* in the human frontal cortex (Additional file 2: Figure S2C).

**Figure 5:**
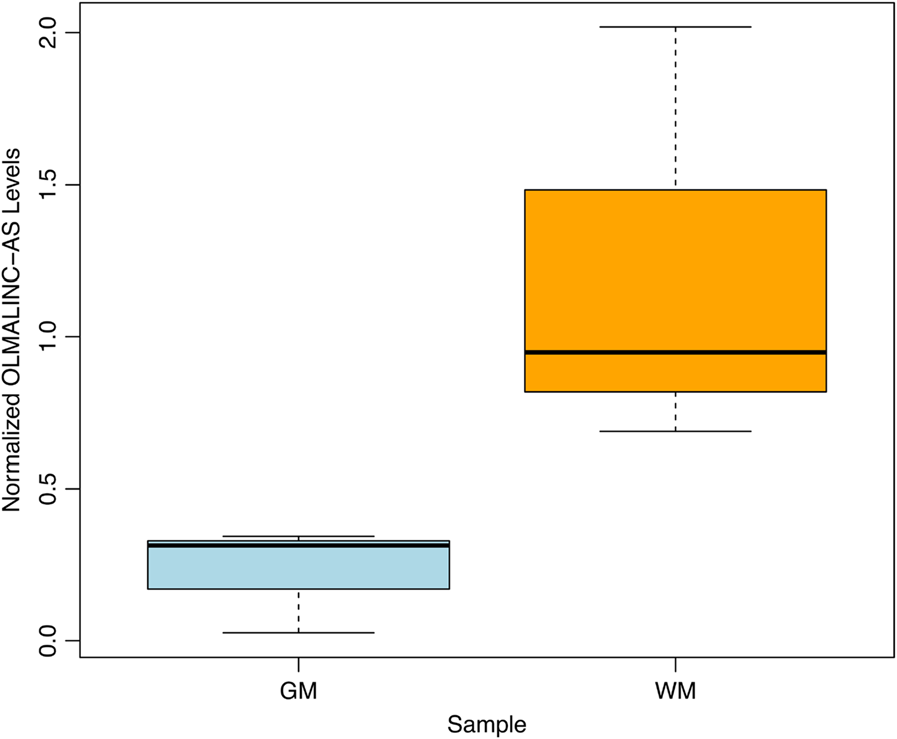
**Quantification of** ***OLMALINC-AS*** **in GM and WM of the frontal cortex.** This boxplot shows *OLMALINC-AS* was up-regulated 11.7-fold in WM (p-value = 0.07).

To further explore the pattern of *OLMALINC-AS* expression in specific histological structures of the cortex, we quantified the levels of this transcript in GM and WM samples using RT-qPCR. Figure 5 shows 11.7-fold up-regulation (p-value = 0.07) of *OLMALINC-AS* in WM. This fold-change did not reach the p-value cut-off level for statistical significances.

### *OLMALINC* knockdown in oligodendrocytes and neurons

To provide insight into the influence of *OLMALINC* on gene expression and regulation of transcription in cortical tissue, we performed RNAi-driven silencing of *OLMALINC* in the human MO3.13 oligodendrocytes and SK-N-SH neurons. The efficiency of the down-regulation of *OLMALINC* expression in the two cell lines was estimated by RT-qPCR. We were able to specifically reduce *OLMALINC* levels in oligodendrocytes and neurons by 4.5- and 3.5-fold, respectively (p-values < 0.05) (Additional file 6: Figure S6).

Next, to assess the impact of *OLMALINC* depletion at the level of the whole transcriptome, we performed RNA-Seq analysis on the *OLMALINC* silenced cells. In MO3.13 oligodendrocytes, 81 genes were up-regulated and 41 were down-regulated (Additional file 7: Table S1). Notably, RNAi-driven reduction of *OLMALINC* expression led to twice as many genes being up-regulated as down-regulated in these cells.

In SK-N-SH neurons, 29 genes showed a significant increase in expression after knockdown of both *OLMALINC* isoforms*,* whereas 17 genes were down-regulated (Additional file 8: Table S2). Again, amongst the genes affected by *OLMALINC* knockdown, nearly twice as many genes showed increased rather than decreased expression in neurons.

### *OLMALINC* knockdown affects genes regulating cell activation and membrane signaling in oligodendrocytes

**Figure 6:**
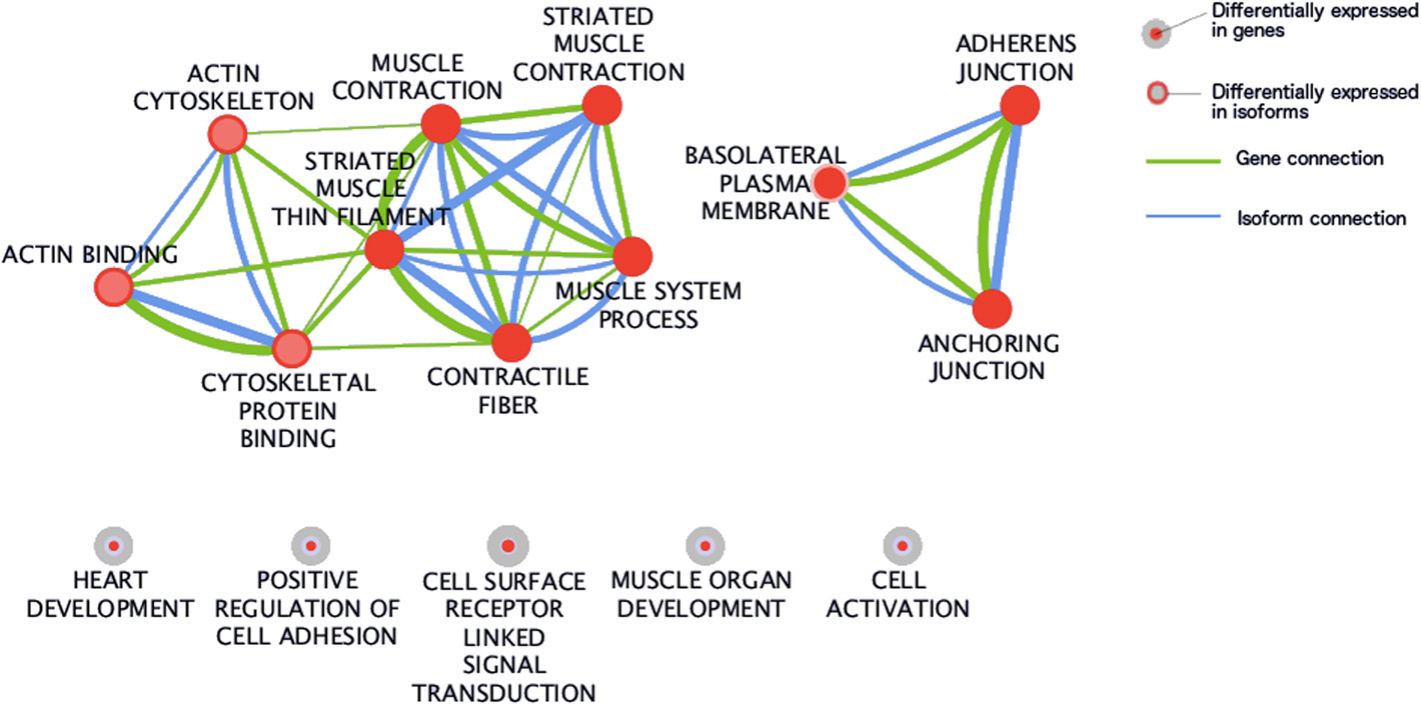
**Enrichment map of the Gene Ontology clusters derived from the DEGs and DEIs in oligodendrocytes following silencing of** ***OLMALINC*** **.** The size of the node relates to the number of genes in each term. Lack of branching in the bottom clusters indicates that genes contributing to these clusters are not present in other clusters and thus are unique for particular pathway.

Gene ontology analysis of differentially expressed genes (DEGs) and differentially expressed isoforms (DEIs) (Additional file 9: Table S3) in oligodendrocytes revealed two superclusters of genes and isoforms that were involved in the regulation and maintenance of cytostructure and cellular adhesion. Moreover, there were four individual clusters related to heart development, cell activation, cell surface receptor-linked signal transduction and positive regulation of cell adhesion (Figure 6). Overall 56 DEGs and DEIs were clustered in the pathway analysis, comprising 34% of the 162 unique DEGs and DEIs that underwent analysis.

**Figure 7:**
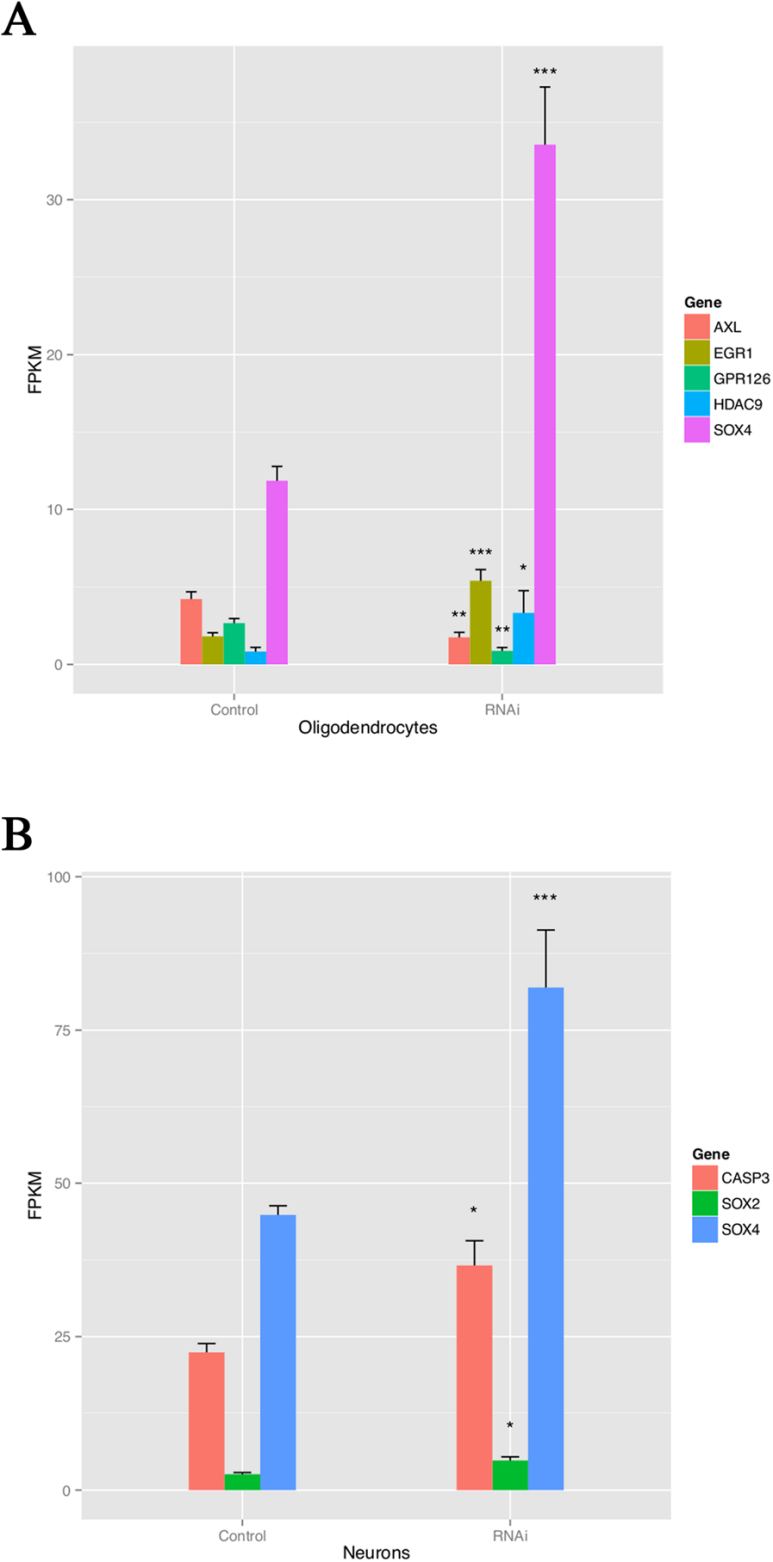
**Expression levels of selected DEGs in** ***OLMALINC*** **-depleted oligodendrocytes (A) and neurons (B).** In oligodendrocytes the *EGR1, HDAC9* and *SOX4* were all up-regulated after RNAi of *OLMALINC. AXL* and *GPR126* was down-regulated in oligodendrocytes after RNAi of *OLMALINC.* The expression levels are in fpkm as calculated by RNA-Seq. In neurons *CASP3, SOX2* and *SOX4* were up-regulated after RNAi of *OLMALINC*. Level of significance: *q-value < 0.05, **q-value < 0.02, ***q-value < 0.01.

Interestingly, amongst the top 25 genes that were up- and down-regulated as a result of *OLMALINC* silencing in oligodendrocytes (Additional file 7: Table S1), 12 genes contributed to the pathway clusters that were linked to the regulation of cell activation and cell surface receptor-linked signal transduction. In contrast, only 2 of the top 25 DEGs contributed to the cytostructure supercluster, and none contributed to the cell adhesion supercluster (Table 1). Several genes of the subset, that are shown in Table 1, caught our attention due to their involvement in the physiology of oligodendrocytes. The expression profiles for these selected genes are shown in Figure 7A.

**Table 1:** **Expression levels for selected DEGs enriched in gene ontology analysis following** ***OLMALINC*** **silencing in oligodendrocytes**

**Gene**	**Chr.**	**Description**	**GO Clusters**	**FPKM Control**	**FPKM RNAi**	**Fold Change**	**q-value**
*SAA1*	chr11	Serum amyloid A1	Cell activation	12.64	2.20	−5.73	0.0014
*IL7R*	chr5	Interleukin 7 receptor	Cell activation, cell surface receptor linked signal transduction	1.60	0.42	−3.83	0.0194
*APLN*	chrX	Apelin	Cell surface receptor linked signal transduction	17.51	5.38	−3.25	2.99E-07
*GPR126*	chr6	G protein-coupled receptor 126	Cell surface receptor linked signal transduction	2.67	0.88	−3.04	0.0116
*CXCL5*	chr4	Chemokine (C-X-C motif) ligand 5	Cell surface receptor linked signal transduction	8.84	2.97	−2.98	4.01E-04
*AXL*	chr19	AXL receptor tyrosine kinase	Cell surface receptor linked signal transduction	4.23	1.75	−2.42	0.0114
*ANXA1*	chr5	Annexin A1	Cell surface receptor linked signal transduction	4.80	2.06	−2.33	4.41E-04
*COL3A1*	chr2	Collagen, type III, alpha 1	Cell activation, cell surface receptor linked signal transduction	380.21	191.13	−1.99	0.0396
*SOX4*	chr6	SRY (sex determining region Y)-box 4	Cell activation, cell surface receptor linked signal transduction	11.85	33.56	2.83	6.13E-11
*EGR1*	chr5	Early growth response 1	Cell activation	1.81	5.41	2.99	3.92E-06
*HDAC9*	chr7	Histone deacetylase 9	Cell activation	0.83	3.33	4.02	0.0486
*WIPF3*	chr7	WAS/WASL interacting protein family, member 3	Cytostructure supercluster	0.79	3.44	4.37	4.09E-08
*TRIM63*	chr1	Tripartite motif containing 63, E3 ubiquitin protein ligase	Cytostructure supercluster	0.35	1.59	4.62	0.0128
*WNT11*	chr11	Wingless-type MMTV integration site family, member 11	Cell surface receptor linked signal transduction	0.13	0.93	7.38	0.0439

Chr. - chromosome.

The histone deacetylase 9 (*HDAC9*) was up-regulated 4.1-fold, after knockdown of *OLMALINC*. The protein product of this gene belongs to class II Hdacs and is expressed mainly in post-mitotic, mature neurons in the murine cerebral cortex [9,10]. Studies of *HDAC9* have revealed its association with medulloblastoma [11] and schizophrenia [9].

SRY (Sex-Determining Region Y)-Box 4 (*SOX4*) was up-regulated 2.8-fold in *OLMALINC*-depleted oligodendrocytes. SRY (Sex-Determining Region Y) (Sox) proteins of group C are strongly expressed in the developing nervous system and have been associated with the maturation of neurons and glia. Prolonged *SOX4* expression in cells of the oligodendrocyte lineage is incompatible with the acquisition of a fully mature phenotype, which indicates that the presence of *SOX4*, and possibly SRY (Sex-Determining Region Y)-Box 11 (*SOX11*), in oligodendrocyte precursors may normally prevent premature differentiation. *SOX4* transgenic mice develop the full spectrum of phenotypic traits that are associated with severe hypomyelination during the first postnatal weeks. In these mice, myelin gene expression is severely reduced, and myelin dramatically thinned in several central nervous system (CNS) regions [12]. *SOX4* expression counteracts the differentiation of radial glia and must be down-regulated before full maturation can occur [13].

G protein-coupled receptor 126 gene (*GPR126*) was down-regulated 3.1-fold in RNAi-treated MO3.13 oligodendrocytes. In Schwann cells, *GPR126* controls proper development and myelination [14]. A mutation in *GPR126* causes severe congenital hypomyelinating peripheral neuropathy in mice and the expression of differentiated Schwann cell markers [15].

AXL receptor tyrosine kinase (*AXL*) is a member of the Tyro3-Axl-Mer (TAM) receptor tyrosine kinase subfamily and was down-regulated 2.5-fold in our RNAi experiments. The TAM receptors are widely expressed in the nervous system, including oligodendrocytes [16]. It has been shown that growth arrest-specific 6 (Gas6), a ligand for the tyro3/Axl/Mer (TAM) receptors, can affect the severity of demyelination in mice, and a loss of signaling via Gas6 leads to decreased oligodendrocyte survival and increased microglial activation during cuprizone-induced demyelination [17]. Gas6 significantly increased myelination in a dose-dependent manner, suggesting that TAM receptor signaling could be directly involved in myelination by oligodendrocytes [18].

Early growth response gene (*EGR1*) is a zinc-finger transcription factor that was 3-fold up-regulated in *OLMALINC*-silenced oligodendrocytes. *EGR1* down-regulation is critical for oligodendrocyte progenitor cell differentiation, and the gene was recently included in a de-repression model of oligodendrocyte lineage progression that relied on the concurrent down-regulation of several inhibitors of differentiation [19]. The expression pattern of selected genes, described above, was confirmed in an independent *OLMALINC* silencing experiment using individual siRNAs followed by RT-qPCR analysis (Additional file 10: Figure S7).

### The cell proliferation pathway is affected by knockdown of *OLMALINC* in neurons

GO enrichment analysis of genes whose expression had been affected by *OLMALINC* knockdown in neurons grouped GO terms for cell proliferation into a single cluster. This cluster was composed of 11 genes that comprised 24% of the all DEGs resulting from *OLMALINC* knockdown in neurons (Additional file 11: Table S4). Several genes within this subset have been previously described to be involved in neuronal biology.

SRY (Sex-Determining region Y)-box 2 gene (*SOX2*) was 1.8-fold up-regulated following *OLMALINC* knockdown. *Sox2* plays a role in the maturation and survival of embryonic and adult neurons, and *Sox2* expression is high in undifferentiated neurons, but declines upon differentiation [20]. *Sox2* deficiency results in decreased precursor cell proliferation and the generation of new neurons in adult mouse neurogenic regions [21].

Caspase-3 (*CASP3*) was up-regulated 1.6-fold following knockdown of *OLMALINC. CASP3* is a key mediator of apoptosis in neuronal cells. The functions of non-apoptotic caspase-3 in neuronal cells include synaptic plasticity, dendrite pruning, as well as learning and memory processes [22].

Finally, *SOX4*, which was up-regulated 1.8-fold in neurons, promotes neuronal differentiation and has a central regulatory role during neuronal maturation. It mechanistically separates cell cycle withdrawal from the establishment of neuronal properties [23]. Figure 7B presents differences in the expression levels of the *SOX2*, *CASP3* and *SOX4* genes as a result of *OLMALINC* silencing.

## Discussion

This study shows that *OLMALINC* is overexpressed in the human brain compared to peripheral tissue and that WM is a major source of this overexpression. Moreover, silencing of *OLMALINC* in oligodendrocytes and neurons affects the expression of functionally related gene sets. Furthermore, this lincRNA is co-expressed with an antisense counterpart, *OLMALINC-AS*, which shows a similar expression pattern, i.e., up-regulation in WM compared to GM. Such co-expression of sense and antisense RNAs with overlapping loci has been previously observed in the case of the *BACE1* mRNA and its natural antisense non-coding partner *BACE1-AS* [24]. Comparative analysis of the *OLMALINC* sequence demonstrates its unique expression in primates.

Recent genome-wide surveys on lincRNAs allow us to draw some general observations about this particular RNA species [25]. First, it has been suggested that most lincRNAs are expressed at low levels, usually below 2 fpkm. In contrast to this observation, *OLMALINC* and its antisense counterpart, *OLMALINC-AS*, remain at levels of at least 16.2 fpkm and reach 71.5 fpkm in WM. Moreover, our meta-analysis of *OLMALINC* expression in peripheral tissues revealed at least 1.9-fold higher expression of this transcript in brain when compared to 15 other cell types of the body. Indeed, lincRNAs have also been shown to exhibit tissue-specific patterns in model organisms such as zebrafish [26]. In particular, the overexpression of *OLMALINC* in the WM corroborated the outcome of *OLMALINC* silencing, which affected a number of genes involved in oligodendrocyte maturation (see below).

Our comparative analysis of the *OLMALINC* sequence with vertebrate genomes revealed a remarkably high degree of conservation in great ape and old world monkey genomes (Figure 3). In all other mammal species examined only homology with exon 1 was observed. There was also evidence for conservation of the region 5′ of exon 1, which was detected as far back as opossum, and even platypus (Figure 3). Although all exons are present in old world monkeys, detectable expression of *OLMALINC* (and its antisense) was only observed in great apes, with exon 3 under expressed outside of humans (Additional file 4: Figure S4). Loss of expression of exons 2 and 3 in gorilla must have occurred after divergence from the human/chimp lineage.

These findings suggests that the building blocks for *OLMALINC* exon 1 are ancient (>200 mya, being common to all mammals), whereas exons 2 and 3 only arose in the ancestor of old world moneys (~30-45 mya) [27]. The evolution of exons 2 and 3 was followed by the expression of *OLMALINC* in at least the Hominidae ancestor (>17 mya), with further specialization (high expression of exon 3) only humans (<6 mya). Although the original building blocks of *OLMALINC* appear ancient, its recently evolved expression suggests that it is a young gene. Indeed, less than 6% of zebrafish lincRNAs have detectable sequence conservation with mouse or human lincRNAs [28]. Moreover, merely 12% of the human and mouse lincRNAs are conserved in other species [29,30].

It has been previously suggested that lincRNAs tend to act in *cis*, thus affecting genes located in a 10-300-kb vicinity of the particular lincRNA locus [31,32]. Interestingly, we have not observed elevated expression of the protein-coding genes located in the 300-kb vicinity of the *OLMALINC* locus (data not shown), which is in contrast one study [33], but corroborates observations of others showing that only 3% of the human lincRNAs have expression profiles correlated with their neighboring genes [34].

Comparative analysis of the *OLMALINC* sequence corroborated earlier observations on its fast evolution [29,30]. Indeed, *OLMALINC* is only conserved within primate genomes, and its homology abruptly drops for rodents and other non-primate vertebrates. The presence of the human-specific exon 3 in the *OLMALINC-002* isoform further supports the thesis that lincRNAs belong to rapidly evolving segments of the primate genome [35].

Along with metastasis associated lung adenocarcinoma transcript 1 (*MALAT-1*) [36] and polyadenylated nuclear RNA (*PAN*) [37], *OLMALINC* belongs to the exceptionally abundantly expressed lincRNAs. High expression levels of some lincRNAs may facilitate their *trans* function as a decoy to titrate proteins from their potential targets, as reported for the growth arrest-specific 5 (*Gas5*) and P21 associated ncRNA DNA damage activated (*PANDA*) lincRNAs [38,39]. Hence, preservation of a titration mechanism will require high numbers of lincRNA molecules interacting with numerous proteins [28].

The RNAi silencing efficiency of *OLMALINC* was less effective in neurons than in an oligodendrocytic cell line, and this could be a result of cell type-specific differences in the uptake capacity of the siRNA between neurons and oligodendrocytes [40]. Second, the initial levels of *OLMALINC* were much below its expression level in oligodendrocytes, which further led to diminution of the silencing effect in neurons.

Silencing of *OLMALINC* expression in oligodendrocytes affected the expression of genes, such as *SOX4*, *GPR126* and *EGR1*, which are involved in the differentiation of these cells from the neuronal stem lineage and in the control of myelination processes. Several non-coding RNAs (ncRNA) have been shown to exhibit dynamic expression patterns during neuronal and oligodendrocyte lineage specification, neuronal-glial fate transitions, and myelination, and they include ncRNAs associated with differentiation-specific nuclear subdomains, such as *Gomafu* and *Neat1,* ncRNAs associated with developmental enhancers, and genes encoding important transcription factors and homeotic proteins [41,42]. Up-regulation of the deacetylase 5 and 9 genes (Additional file 7: Table S1) by depletion of *OLMALINC* further underpins the previous observations of the involvement of lincRNAs in chromatin remodeling-controlling cellular differentiation, as has been shown in lineage-specific gene expression programs in mouse embryonic stem cells [43].

The number of genes whose expression was affected by *OLMALINC* silencing remains similar to the average of 175 protein-coding genes that changed their expression pattern in 137 individual lincRNA knockdowns in Guttman’s study (2011). Moreover, changes in expression were characterized by up- and down-regulation, suggesting a versatile impact of *OLMALINC* on transcription that leads to the activation or repression of transcription. This remains in contrast to previous suggestions that the action of lincRNAs mainly leads to repression of gene expression [44].

## Conclusions

Our evidence indicates that the recently evolved *OLMALINC* is a primate specific transcript that is a major contributor to the maintenance of oligodendrocyte maturation. This conclusion remains in line with previous observations that lincRNAs are involved in development of the nervous system [45]. However, it should be emphasized that further characterization of *OLMALINC* function will require systematic studies, including defining all protein complexes with which the lincRNA possibly interacts, determining where these protein interactions assemble on the RNA, and ascertaining whether they bind simultaneously or alternatively. Moreover, understanding how *OLMALINC*–protein or –DNA interactions give rise to specific patterns of gene expression will require determination of the functional contribution of each interaction and possible localization of the complex to its genomic targets.

## Methods

### Brain tissue and RNA extraction

Samples representing the GM and WM of the human superior frontal gyrus (SFG) were obtained from the Sydney Brain Bank following ethical approval from the Human Research Ethics Committee of the University of New South Wales. The GM and WM tissues were obtained from three individuals (aged 79, 94 and 98) without significant neuropathology. The post mortem interval (PMI) of the samples ranged from 8–24 h, and the pH ranged from 5.77-6.65. For RT-qPCR experiments, another two SFG samples were used that were matched to the previous samples regarding age, PMI and pH.

Total RNA was extracted from approximately 100 mg for each case using Qiagen’s RNeasy Lipid Tissue RNA Extraction Kit. The RNA integrity numbers (RINs) were determined using an Agilent 2100 BioAnalyzer RNA Nano Chip. The RIN values ranged from 4.9-7.2. This RIN range was previously shown to have little effect on relative gene expression ratios [46].

### Cell lines

MO3.13 oligodendrocytes and SK-N-SH neurons were obtained from the ATCC (Manassas, VA) and were cultured in 12-well plates in Dulbecco’s modified Eagle’s medium containing 10% fetal calf serum, 2 mM glutamine, 100 IU/ml penicillin, and 100 μg/ml streptomycin at 37°C in humidified air containing 5% CO_2_. Cell culture media and additives were obtained from Invitrogen (Melbourne, Australia) unless stated otherwise.

### Reverse transcription and PCR

Using total RNA samples previously used for the RNA-Seq analysis [5], the *OLMALINC-002, −003* and *-AS* transcripts were reverse transcribed and amplified using the Qiagen One-Step RT-PCR kit. The forward and reverse primers used to amplify *OLMALINC-002* were TGTGGTACTAAGCTTGACAGC and TCATAGGTGGATCTCCTCACG; for *OLMALINC-003,* TAGACCTTGCTAACCAGGACG and TGGTATCAGTTAGCGTGGGGC; and for *OLMALINC-AS,* CCCGAGATTCTTTGTGGGCT and CTCTCCCACCACACACCAC. Standard RT-PCR conditions, as recommended by Qiagen, were used. PCR products of 560, 1100 and 280 bp were purified and Sanger sequenced.

### Quantitative PCR

Primers were designed for the *OLMALINC*, *OLMALINC-AS* and proteasome (prosome, macropain) subunit beta type 4 (*PSMB4*) genes. For quantification of *EGR1*, *HDAC9*, *SOX4*, *GPR126* and *AXL* expression in MO3.13 oligodendrocytes primers were purchased from Qiagen. We used *PSMB4* as a housekeeping gene, as described previously [47]. The *PSMB4* forward and reverse primers were ordered from Qiagen (HS_PSMB4_1_SG QuantiTect primer assay). The sequence for the *OLMALINC* forward primer was GACTCCTTTGGGAGACCAGTG, and that of the reverse primer was AGGTCACAGGGGATTTGATGG. The *OLMALINC* primers spanned the fragment of the transcript that was common for the *OLMALINC-002* and −*003* isoforms. The sequence for the *OLMALINC-AS* forward primer was GTCACTGGGGAGAACGTGAC, and that for the reverse primer was CTCTCCCACCACACACCAC. The *OLMALINC-AS* primers were unique for the *–AS* transcript. All of the primers used for RT-qPCR had an efficiency of between 90%-110%. The RNA samples were reverse-transcribed using the Qiagen QuantiTect Reverse Transcription (RT) Kit, and the gene expression was quantified using the QuantiTect SYBR green PCR master mix (Qiagen). The PCR reaction was performed on a Rotor-Gene 6000 (Qiagen) using three independent GM and WM samples and MO3.13 oligodendrocytes, and each reaction was performed in triplicate. *PSMB4* was used to normalize the results from each RT-qPCR run to reduce batch effects and correct any variation in template input [48]. Expression levels were calculated using 2^-ΔCt^ and fold-change was calculated using the 2^-ΔΔCt^ method [49]. Statistically significant changes in gene expression were assessed using the R project for statistical computing (www.r-project.org).

### RNA interference

RNAi knockdown of *OLMALINC* was carried out using four commercially prepared siRNA oligonucleotides (Qiagen) specific to different fragments of the *OLMALINC* transcript. Briefly, MO3.13 and SK-N-SH cells were seeded at 40% confluence in 6-well plates in antibiotic-free media and equimolar mix of four oligonucleotides was transfected using Lipofectamine 2000 and Opti-MEM I (Invitrogen) following the manufacturer’s protocol. For validation experiments MO3.13 oligodendrocytes were transfected with two individual siRNAs used previously in the four siRNA mix. Transfection efficiency was confirmed by substituting the siRNA with fluorescently labelled BLOCK-iT reagent (Invitrogen), using the same transfection procedure and this confirmed >95% transfection efficiency was achieved as assessed by fluorescence microscopy.

### RNA isolation, library preparation and sequencing

After 48 hrs of culture total RNA was isolated using RNeasy Mini Kit (Qiagen) followed by RNase-free DNase treatment to remove traces of genomic DNA. The RNA quality of the total RNA was assessed using the Agilent 2100 BioAnalyser RNA Nano Chip and the RIN values ranged between 8.0 and 9.0. Six RNA samples (three MO3.13 and three SK-N-SH replicates) were prepared for sequencing according to the Illumina TruSeq RNA sample preparation guide and subjected to 100 bp paired-end sequencing using Illumina HiSeq1000.

### Meta-analysis of Illumina expression data of non-brain tissues

Meta-analysis of Illumina BodyMap2 transcriptome files was carried out to determine the expression distribution of *OLMALINC* across tissues at the gene level. The BodyMap2 project was carried out by Illumina to provide a sample RNA-Seq dataset of 16 individual and mixed tissues. A second analysis was performed on five independent RNA-Seq datasets produced by The Human Protein Atlas (http://www.proteinatlas.org/tissue). The meta-analysis was carried out on Galaxy using the Tuxedo protocol [13], and files from Illumina’s BodyMap2 project and the Human protein Atlas project were imported into Galaxy from the NCBI sequence read archive (SRA) (project accession number: ERP000546, http://www.ncbi.nlm.nih.gov/sra/?term=ERP000546, project accession number: ERP003613, http://www.ncbi.nlm.nih.gov/sra/?term=ERP003613). The selected SRA file reads were assembled with TopHat, which utilizes Bowtie to align short sequence reads to the *H. sapiens* reference genome (build hg19). The default setting for TopHat was used. Cufflinks was then used to assemble the aligned reads into individual transcripts by inferring splicing structure and provided a minimal number of predicted transcripts through parsimonious assembly. Cufflinks also normalized the read count of each input file to allow for calculation of the relative abundance of each transcript in fpkm. To guide the assembly process, the iGenomes UCSC hg19 full annotation GTF file was used. These steps were performed for all 16 tissue types that were assessed. Subsequently, the results for each tissue type were merged with the files for the brain transcriptome and the iGenomes reference annotation via Cuffmerge and then passed through Cuffdiff.

### Comparative analysis with vertebrate genomes

The UCSC Genome Browser website (http://genome.ucsc.edu/index.html) contains the reference sequences and working draft assemblies of a large collection of genomes from a variety of organisms. Using the UCSC Genome Browser, the level of sequence conservation of *OLMALINC* across numerous species was analyzed.

### Gene ontology enrichment analysis

The lists of DEGs and DEIs were entered into the Database for Annotation, Visualization and Integrated Discovery (DAVID) (http://david.abcc.ncifcrf.gov/) [50]. DAVID can only utilize annotated genes/isoforms; thus, all un-annotated genes/isoforms and indecisively annotated genes/isoforms were removed from the lists. DAVID tests GO terms for over representation in each of the DEG and DEI lists. The GO terms lost produced by DAVID, were processed using the ‘Enrichment Map’ plug in for ‘Cytoscape’ (http://www.cytoscape.org/) [51]. This produces a visual putput of the text based GO term lists.

### Data access

The sequence data have been submitted to the NCBI Short Read Archive with accession number SRA602249.

## Additional files

Additional file 1: Figure S1. Differential expression of the *OLMALINC* gene and its isoforms as revealed by RNA-Seq analysis of GM and WM samples from the human frontal cortex. **(A)** Expression levels of the *OLMALINC* gene in GM and WM. **(B)** Expression levels of the *OLMALINC-002* and −*003* isoforms in GM and WM; bars represent SD. Level of significance: **q-value< 0.02, ***q-value<0.01. Additional file 2: Figure S2. Sequence alignment of Sanger sequenced RT-PCR *OLMALINC* products with sequences derived from RNA-Seq data. The query sequence is the sequence derived from Sanger sequencing and the subject sequence is derived from RNA-Seq data. A. Sequence alignment of *OLMALINC-002*. B. Sequence alignment of *OLMALINC-003* C. Sequence alignment of *OLMALINC-AS.* The letter N marks nucleotides where sequencing failed to develop a consensus sequence. Additional file 3: Figure S3. Homology detected to *OLMALINC* in the chimpanzee, gorilla, orangutan, rhesus monkey, marmoset and opossum genomes with blat searches on the UCSC genome browser. All homologies are displayed relative to the *SCD* gene. In the opossum genome (reverse orientation) homology to exon 1 was not detected with blat searches, but was detected in the multiZ alignments track. In rhesus monkey, exon 3 is not detected in the correct position with balt searches, but is detected with blastz. Exon 1 homologies are boxed in red, exon 2 in green, and exon 3 in blue. Basewise conservation calculated by PhyloP across 100 vertebrates shows a peak in conservation upstream of *OLMALINC* exon 1 indicated a possible promoter region. Additional file 4: Figure S4. Read coverage from male brain RNA-seq data across the *OLMALINC* homologous regions in human, chimpanzee, gorilla, orangutan, rhesus monkey and opossum. Outside of great apes there is no evidence for expression of any exon. Exon 1 homologies are boxed in red, exon 2 in green, and exon 3 in blue. Read depth bedGraph files were available for human, gorilla, rhesus monkey and opossum [11]. For chimpanzee and orangutan, RNA-seq reads [12] were mapped with TopHat v2.0.9 with the command line options: tophat -p 4 -a 8 -i 40 -m 1 -I 1000000 --coverage-search --microexon-search. Read depth coverage bedGraph files were generated with the samtools depth utility. Additional file 5: Figure S5. Comparative analysis of *OLMALINC* expression across five tissue samples. The RNA-Seq data sets were taken from the Human Protein Atlas project (http://www.proteinatlas.org/). Again, *OLMALINC* is expressed at its highest level in brain tissue and it is expressed 3-fold higher than liver, the tissue source with the next highest level of expression. The independent dataset confirms the results from Figure 4. The y-axis is expression in fpkm. Additional file 6: Figure S6. Quantification of the *OLMALINC* transcript following its knockdown in oligodendrocytes and neurons using RT-qPCR. *OLMALINC* levels in oligodendrocytes and neurons were reduced by 4.5- and 3.5-fold, respectively (p-values<0.05). N – neurons; O – oligodendrocytes. Additional file 7: Table S1. Full list of differentially expressed genes and isoforms between the oligodendrocyte cell controls and the oligodendrocyte cells that underwent RNAi. Additional file 8: Table S2. Full list of differentially expressed genes and isoforms between the neuronal cell controls and the neuronal cells that underwent RNAi. Additional file 9: Table S3. Enriched gene ontology terms for genes and isoforms differentially expressed in the oligodendrocyte cell line. Additional file 10: Figure S7. RT-qPCR validation of the *EGR1, HDAC9, SOX4, AXL* and *GPR126* genes expression pattern in MO3.13 oligodendrocytes silenced with individual *OLMALINC* siRNAs. *EGR1, HDAC9 and SOX4* genes were up-regulated 4-, 4.4-, 3.6-fold in RNAi-treated oligodendrocytes when compared to control (p-value<0.05), respectively. The *GPR126* and *AXL* genes were down-regulated 3.5- and 7.5-, respectively. Con – control. Additional file 11: Table S4. Enriched gene ontology terms for genes and isoforms differentially expressed in the neuronal cell line.

## Abbreviations

*AXL*: AXL receptor tyrosine kinase
*BACE1*: Beta-site APP-cleaving enzyme 1
*BACE1-AS*: Beta-site APP-cleaving enzyme 1 antisense RNA
*CASP3*: Caspase-3
CNS: Central nervous system
*EGR1*: early growth response 1
Fpkm: Fragments per kilobase of exon per million fragments mapped
*Gas5*: Growth arrest-specific 5 (non-protein coding)
Gas6: Growth arrest-specific 6
GM: Grey matter
*Gomafu*: myocardial infarction associated transcript (non-protein coding)
*GPR126*: G protein-coupled receptor 126
Hdac: Histone deacetylase
*HDAC9*: Histone deacetylase 9
HIF-1alpha: Hypoxia inducible factor 1, alpha subunit (basic helix-loop-helix transcription factor)
ING4: Inhibitor of growth family, member 4
*OLMALINC*: Oligodendrocyte maturation-associated long intervening non-coding RNA
*OLMALINC-AS*: Oligodendrocyte maturation-associated long intervening non-coding RNA
lincRNAs: long non-coding intervening RNAs
lncRNAs: Long non-coding RNAs
LTR: long terminal repeat
*Malat1*: Metastasis associated lung adenocarcinoma transcript 1
*Neat1*: Nuclear paraspeckle assembly transcript 1 (non-protein coding)
ncRNA: Non-coding RNA
nts: Nucleotides
ORF: Open reading frame
*PAN*: Polyadenylated nuclear RNA
*PANDA*: P21 associated ncRNA DNA damage activated
PMI: Post mortem interval
*PSMB4*: Proteasome (prosome, macropain) subunit, beta type, 4
RIN: RNA integrity number
RNAi: RNA-interference
RNA-Seq: RNA-Sequencing
RT-qPCR: Reverse transcription quantitative polymerase chain reaction
*SCD*: Stearoyl-CoA desaturase (delta-9-desaturase)
SFG: Superior frontal gyrus
Sox: SRY (sex-determining region Y)
*SOX2*: SRY (sex-determining region Y)-box 2
*SOX4*: SRY (Sex-determining region Y)-box 4
*SOX11*: SRY (sex-determining region Y)-box 11
TAM: tyro3/Axl/Mer
TFs: Transcription factors
WM: White matter
