# Supplementary figures and images for: High expression of long intervening non-coding RNA OLMALINC in the human cortical white matter is associated with regulation of oligodendrocyte maturation
Source: Mol Brain. 2015 Jan 10;8:2. doi: 10.1186/s13041-014-0091-9 (PMC4302521; doi:10.1186/s13041-014-0091-9)

**A.**

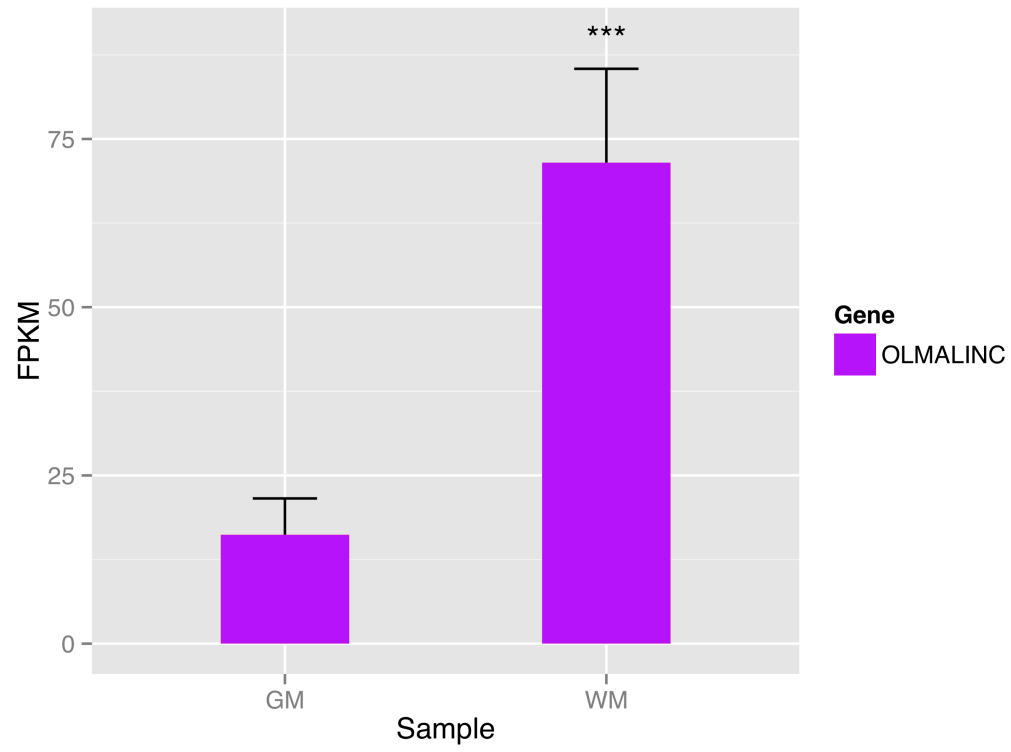

**B.**

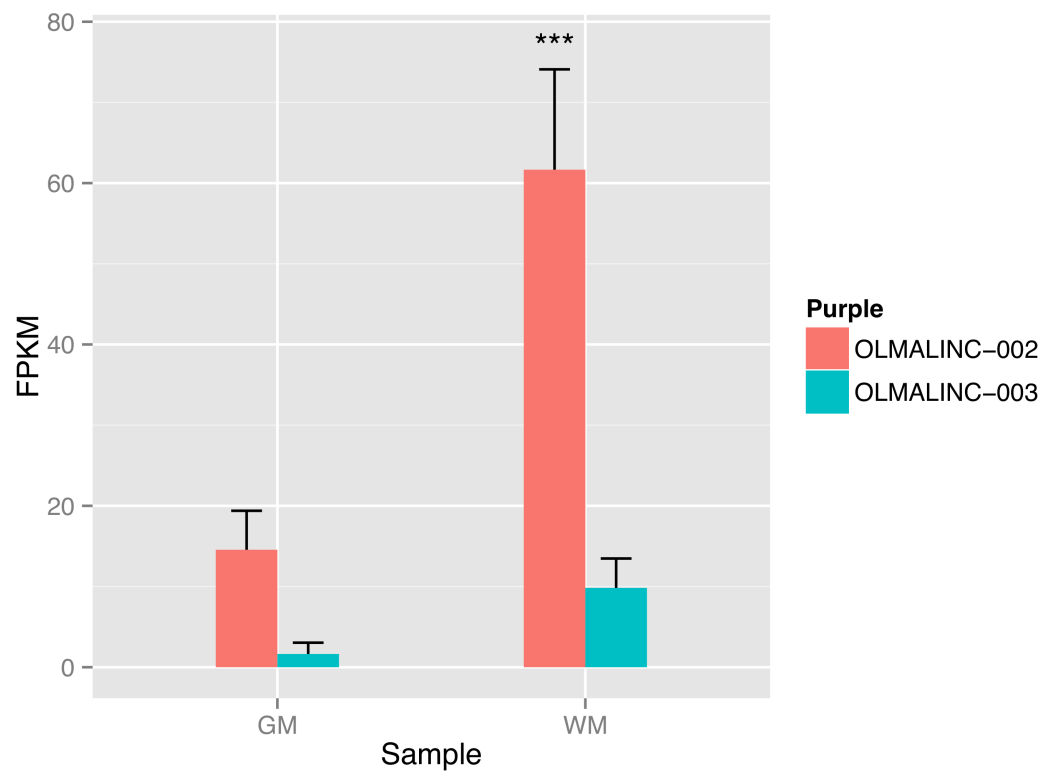

**Suppl. Fig. 1**

Supplement: Additional file 1: Figure S1. — Differential expression of the OLMALINC gene and its isoforms as revealed by RNA-Seq analysis of GM and WM samples from the human frontal cortex. (A) Expression levels of the OLMALINC gene in GM and WM. (B) Expression levels of the OLMALINC-002 and −003 isoforms in GM and WM; bars represent SD. Level of significance: **q-value< 0.02, ***q-value<0.01. [file 13041_2014_91_MOESM1_ESM.pdf]

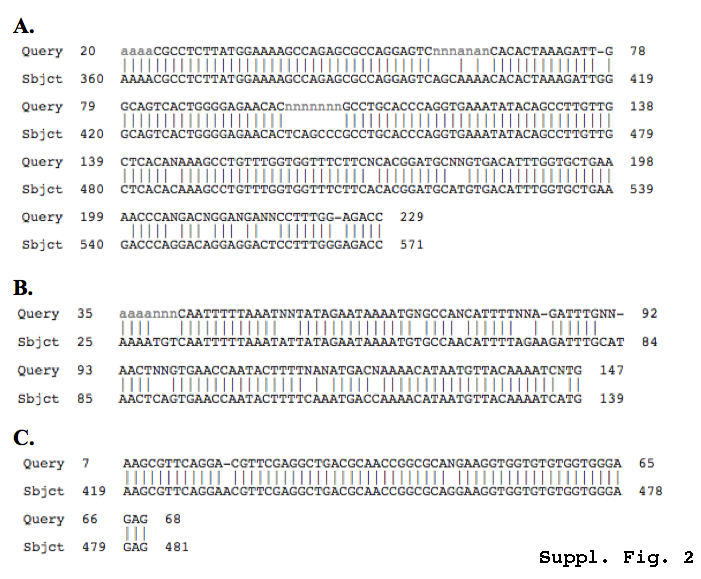

Supplement: Additional file 2: Figure S2. — Sequence alignment of Sanger sequenced RT-PCR OLMALINC products with sequences derived from RNA-Seq data. The query sequence is the sequence derived from Sanger sequencing and the subject sequence is derived from RNA-Seq data. A. Sequence alignment of OLMALINC-002. B. Sequence alignment of OLMALINC-003 C. Sequence alignment of OLMALINC-AS. The letter N marks nucleotides where sequencing failed to develop a consensus sequence. [file 13041_2014_91_MOESM2_ESM.png]

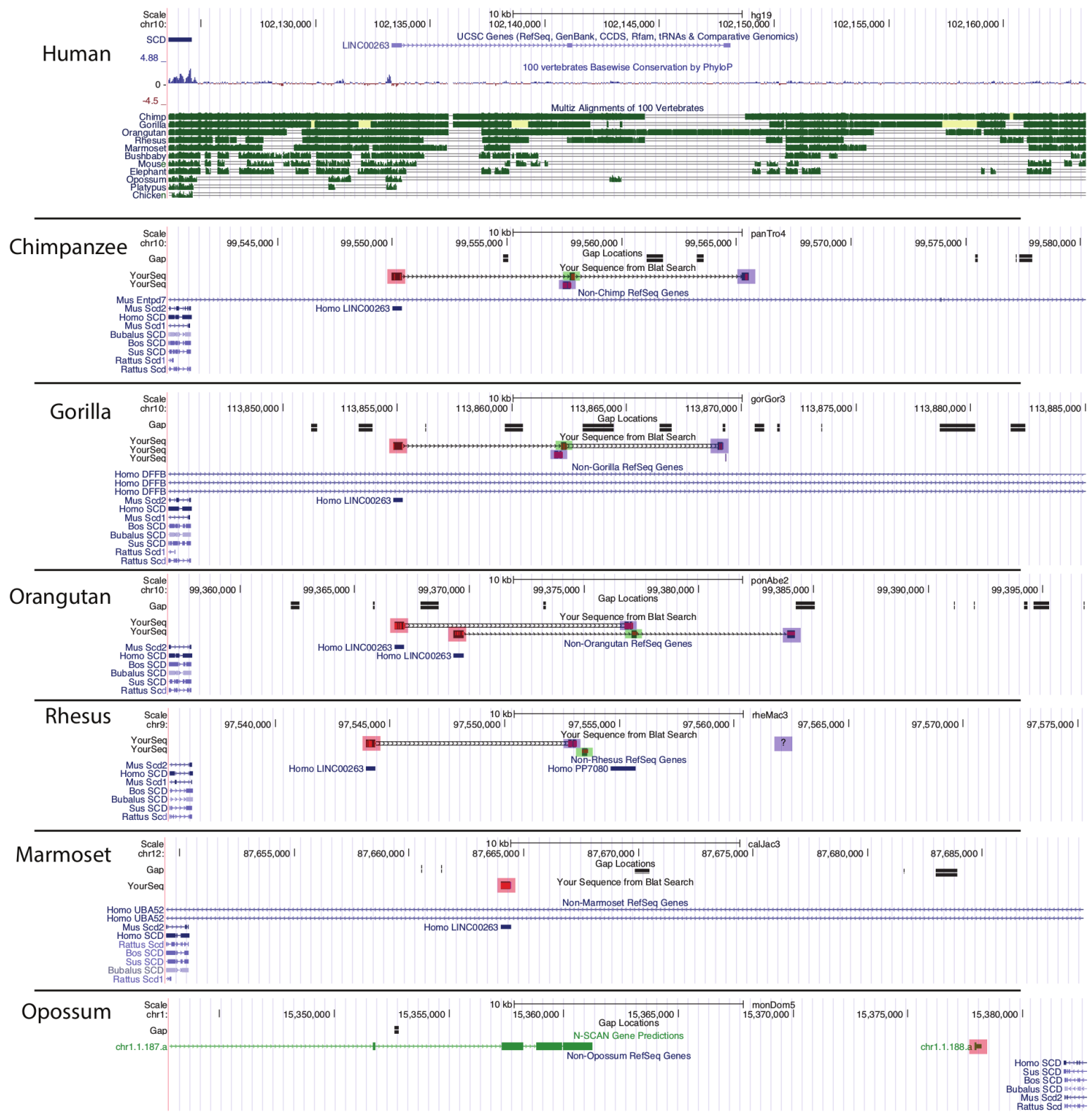

Suppl. Fig. 3

Supplement: Additional file 3: Figure S3. — Homology detected to OLMALINC in the chimpanzee, gorilla, orangutan, rhesus monkey, marmoset and opossum genomes with blat searches on the UCSC genome browser. All homologies are displayed relative to the SCD gene. In the opossum genome (reverse orientation) homology to exon 1 was not detected with blat searches, but was detected in the multiZ alignments track. In rhesus monkey, exon 3 is not detected in the correct position with balt searches, but is detected with blastz. Exon 1 homologies are boxed in red, exon 2 in green, and exon 3 in blue. Basewise conservation calculated by PhyloP across 100 vertebrates shows a peak in conservation upstream of OLMALINC exon 1 indicated a possible promoter region. [file 13041_2014_91_MOESM3_ESM.pdf]

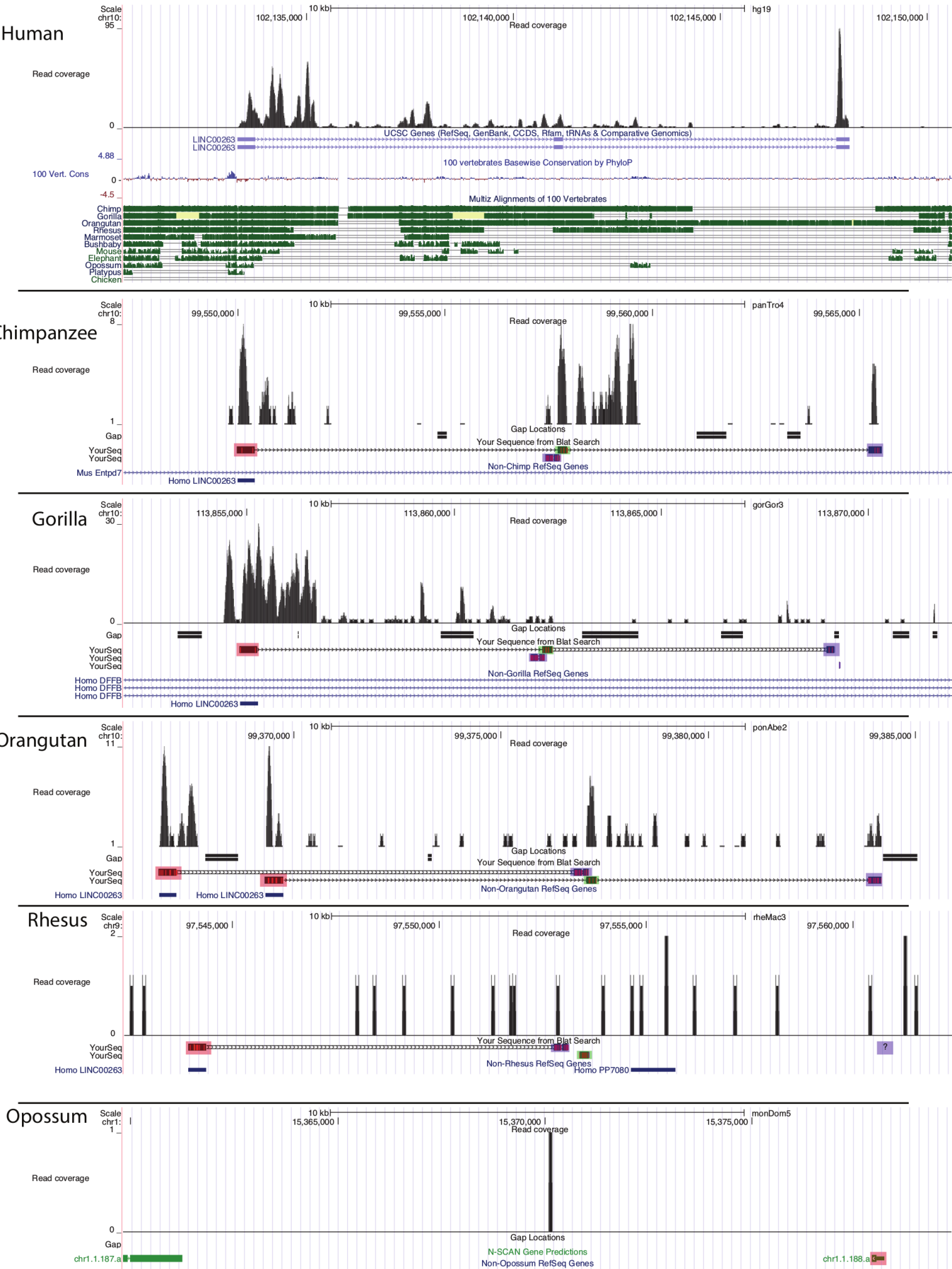

Suppl. Fig. 4

Supplement: Additional file 4: Figure S4. — Read coverage from male brain RNA-seq data across the OLMALINC homologous regions in human, chimpanzee, gorilla, orangutan, rhesus monkey and opossum. Outside of great apes there is no evidence for expression of any exon. Exon 1 homologies are boxed in red, exon 2 in green, and exon 3 in blue. Read depth bedGraph files were available for human, gorilla, rhesus monkey and opossum [11]. For chimpanzee and orangutan, RNA-seq reads [12] were mapped with TopHat v2.0.9 with the command line options: tophat -p 4 -a 8 -i 40 -m 1 -I 1000000 --coverage-search --microexon-search. Read depth coverage bedGraph files were generated with the samtools depth utility. [file 13041_2014_91_MOESM4_ESM.pdf]

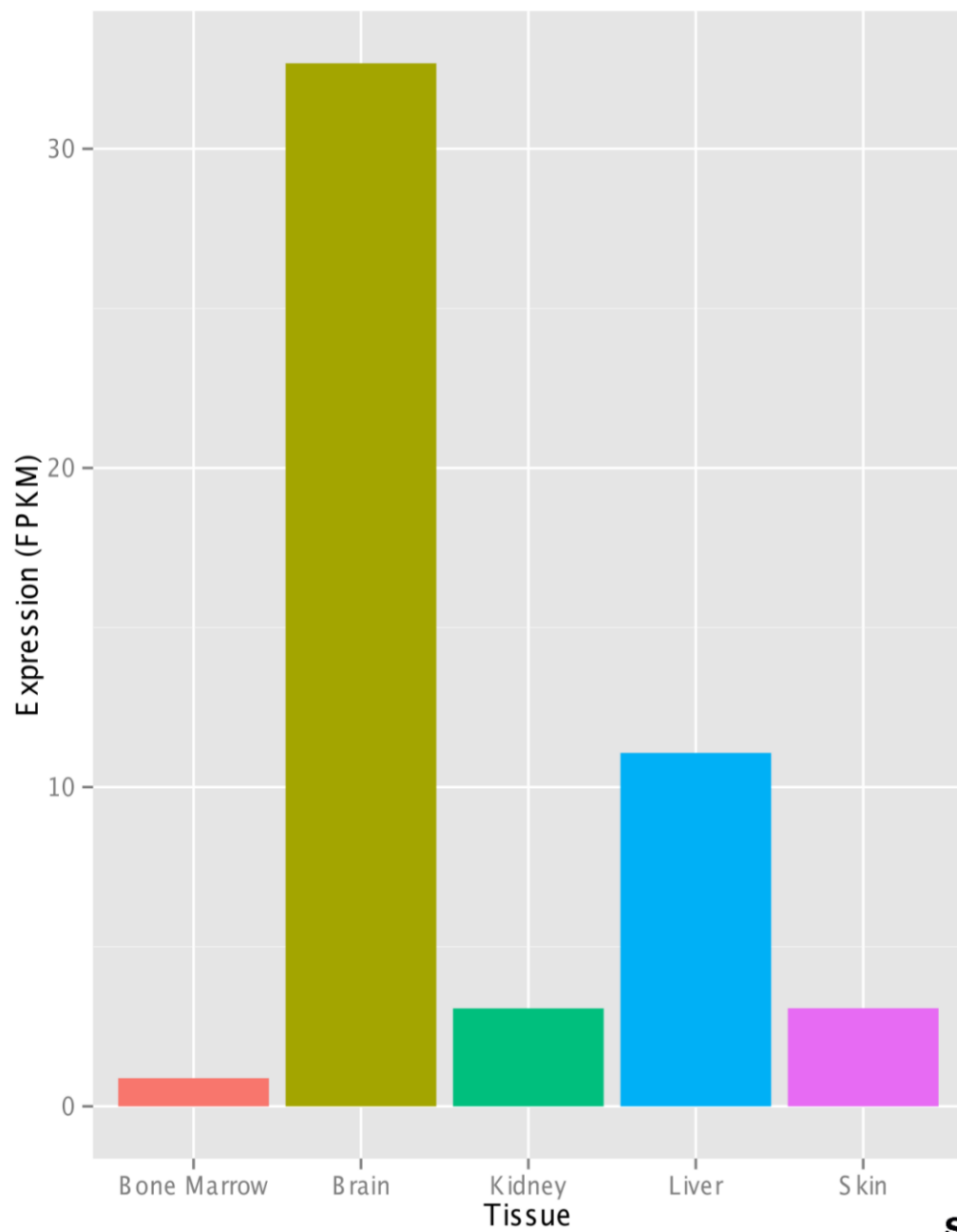

**Suppl. Fig. 5**

Supplement: Additional file 5: Figure S5. — Comparative analysis of OLMALINC expression across five tissue samples. The RNA-Seq data sets were taken from the Human Protein Atlas project (http://www.proteinatlas.org/). Again, OLMALINC is expressed at its highest level in brain tissue and it is expressed 3-fold higher than liver, the tissue source with the next highest level of expression. The independent dataset confirms the results from Figure 4. The y-axis is expression in fpkm. [file 13041_2014_91_MOESM5_ESM.pdf]

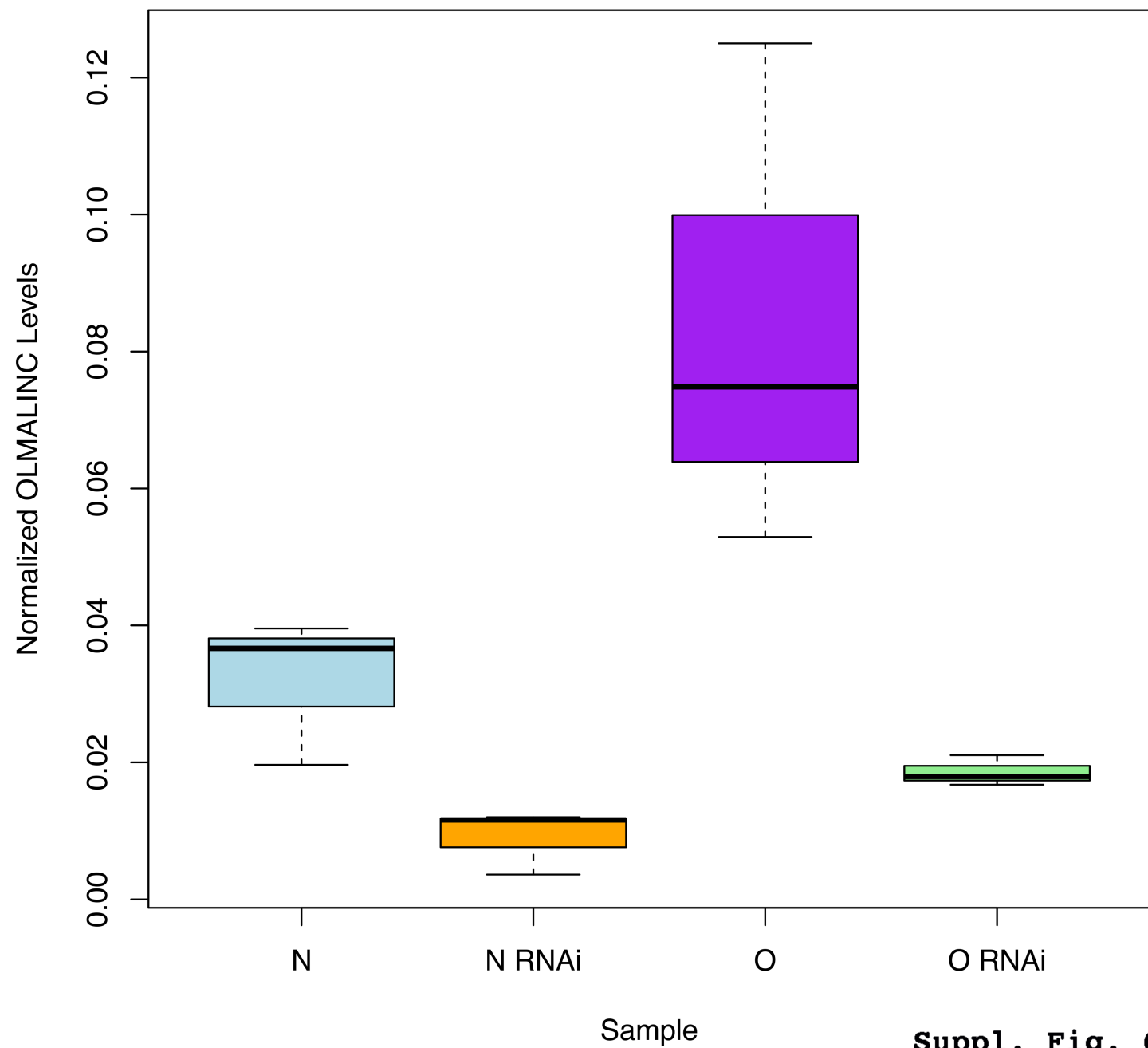

**Suppl. Fig. 6**

Supplement: Additional file 6: Figure S6. — Quantification of the OLMALINC transcript following its knockdown in oligodendrocytes and neurons using RT-qPCR. OLMALINC levels in oligodendrocytes and neurons were reduced by 4.5- and 3.5-fold, respectively (p-values<0.05). N – neurons; O – oligodendrocytes. [file 13041_2014_91_MOESM6_ESM.pdf]

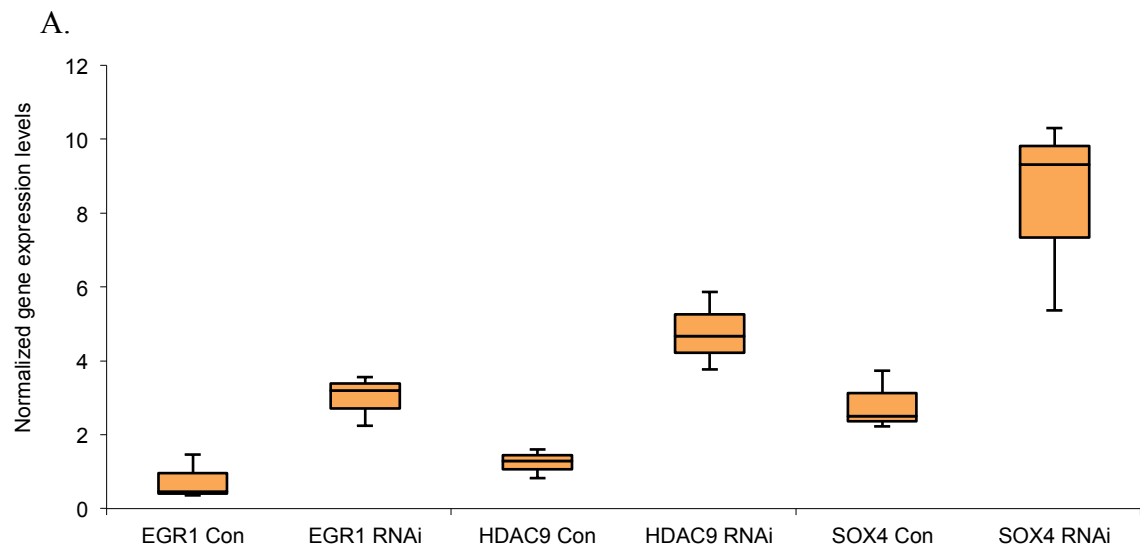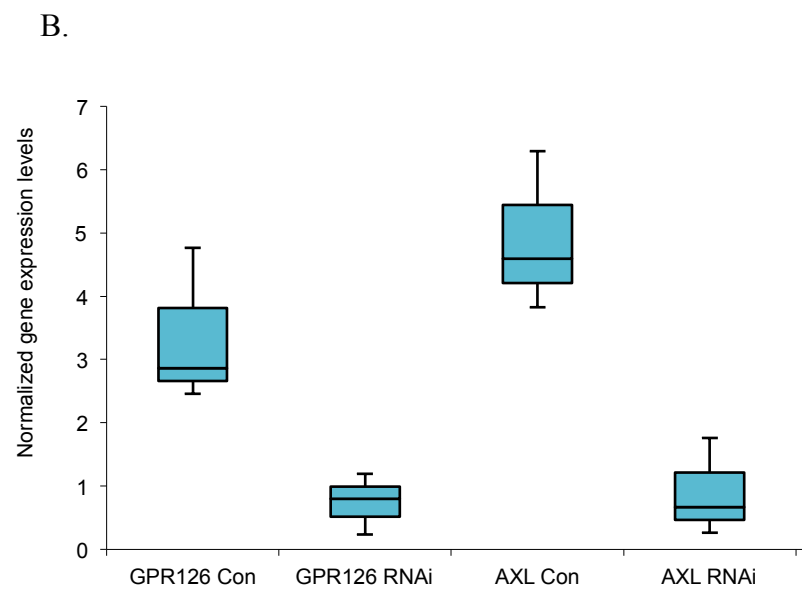

Supplement: Additional file 10: Figure S7. — RT-qPCR validation of the EGR1, HDAC9, SOX4, AXL and GPR126 genes expression pattern in MO3.13 oligodendrocytes silenced with individual OLMALINC siRNAs. EGR1, HDAC9 and SOX4 genes were up-regulated 4-, 4.4-, 3.6-fold in RNAi-treated oligodendrocytes when compared to control (p-value<0.05), respectively. The GPR126 and AXL genes were down-regulated 3.5- and 7.5-, respectively. Con – control. [file 13041_2014_91_MOESM10_ESM.pdf]
